# Free Dispensaries

**Published:** 1880-03

**Authors:** 


FREE DISPENSARIES.—We are glad to see that movements are being made in the principal cities of England and this country to abate, if not altogether check, this absolute robbery of the general practitioner, and we feel the time has arrived for energetic action in the same direction in Baltimore. We know not what percentage of people, able to pay moderate medical fees, visit these so-called charities, but it must be considerable, and unless steps are taken to arrest the evil it will certainly continue to grow apace with the food it feeds upon. With the lights now before us, we might ask *cui bono* of these institutions, were it not that it is seen the medical attendants who have them in charge are benefited by the advertising which such official positions enable them to occupy before the public. Now, while such dispensary attendants are in nowise superior, as a general rule, to their professional neighbors, the fact that they are known to hold such official relations induces a *prima facie* impression on a credulous public that they are such, and that they are consequently better prepared to treat the infirmities to which it is subject. This is not only all wrong, in fact, but unjust in its influence on the business of the general practitioner, and ought to have the proper remedy applied for its abatement at once. We would invite careful attention to the article in this issue from the New York *Medical Record*, on this subject, entitled "The Abuses of Medical Charity," which was received since the foregoing editorial comments were written. It is our purpose to extend whatever aid we can in abating the nuisance to which we refer, and heartily join in the important movement now fully inaugurated in that direction.

